# Peripheral Versus Central Cannulation for Venoarterial Extracorporeal Membrane Oxygenation (VA-ECMO): A Meta-Analysis of Bleeding and Vascular Complications

**DOI:** 10.7759/cureus.101271

**Published:** 2026-01-11

**Authors:** Giorgi Chilingarashvili, Giorgi Maisuradze, Aimal Shah, Franco Campoli, Laura Knittig, Avilash Mondal, Vakhtang Robakidze, Tuy Nguyen, Vien Truong

**Affiliations:** 1 General Internal Medicine, Nazareth Hospital, Philadelphia, USA; 2 Oncology, Tbilisi State Medical University, Tbilisi, GEO; 3 Biomedical Sciences, Georgian American University, Tbilisi, GEO; 4 Internal Medicine, Ayub Medical College, Abbottabad, PAK; 5 Internal Medicine, Nazareth Hospital, Philadelphia, USA; 6 Cardiology, West Virginia University (WVU) Heart and Vascular Institute, Morgantown, USA; 7 Internal Medicine, Tbilisi State Medical University, Tbilisi, GEO; 8 Radiology, Tam Anh General Hospital, Ho Chi Minh City, VNM; 9 Cardiology, The Christ Hospital Health Network, Cincinnati, USA

**Keywords:** cardiogenic shock, central cannulation, limb ischemia, peripheral cannulation, venoarterial extracorporeal membrane oxygenation (va-ecmo)

## Abstract

Extracorporeal membrane oxygenation (ECMO) is increasingly utilized in refractory cardiogenic shock, though the optimal cannulation strategy remains debated, due to divergent vascular and bleeding complications. Peripheral (femoral) access offers ease of deployment but may increase limb ischemia risk, while central (aortic/right atrial) cannulation improves antegrade flow, but carries a higher surgical bleeding burden. We performed a meta-analysis to compare outcomes between peripheral and central cannulation, focusing on major complications, including bleeding, limb ischemia, infection, renal replacement therapy (RRT), and cerebrovascular accidents (CVAs).

We systematically searched MEDLINE, Scopus, and Cochrane CENTRAL from inception through February 2025, excluding overlapping registry-based analyses. The DerSimonian-Laird effects model was applied to compute pooled odds ratios (ORs) with 95% confidence intervals (CIs). Publication bias was assessed using a visual funnel plot and the Egger's and Begg's tests. A leave-one-out sensitivity analysis was conducted to evaluate the robustness of the findings. All analyses were conducted in R statistical software (v4.3.2, R Foundation for Statistical Computing, Vienna, Austria), using the meta, metafor, and dmetar packages.

Fifteen studies were included (N = 2,913). Patients receiving peripheral ECMO were slightly younger (54.8 ± 14.3 vs. 57.0 ± 13.7 years) and more often male (72% vs. 64%). Peripheral cannulation was associated with a lower risk of major bleeding (risk ratio (RR) 0.55, 95% CI 0.43-0.70), but a higher risk of limb ischemia (RR 1.43, 95% CI 1.17-1.75). No significant differences were observed for infection (RR 0.88, 95% CI 0.39-2.01), RRT (RR 1.17, 95% CI 0.66-2.08), or CVA (RR 1.19, 95% CI 0.78-1.83). Sensitivity analyses, using a leave-one-out approach, confirmed the robustness of the findings, yielding nearly identical pooled estimates and indicating that no single study disproportionately influenced the results.

Peripheral venoarterial ECMO (VA-ECMO) cannulation is associated with a significantly lower risk of bleeding, but a higher risk of limb ischemia, compared with central access, with no significant differences observed in infection, RRT, or CVAs. Therefore, the choice between peripheral and central access should be individualized, based on patient-specific risk profiles, particularly balancing bleeding risk against ischemic risk.

## Introduction and background

Cardiogenic shock is a life-threatening condition, characterized by reduced cardiac output in the presence of adequate intravascular volume, resulting in tissue hypoxia [1]. Despite advances in therapy, cardiogenic shock continues to carry mortality rates approaching 50% [2]. Mechanical circulatory support systems have increasingly been used to stabilize hemodynamics in patients with cardiogenic shock. Among them, extracorporeal membrane oxygenation (ECMO) is recommended primarily for refractory cases [2]. While venovenous ECMO depends on relatively stable hemodynamics, venoarterial ECMO (VA-ECMO) provides both cardiac and respiratory support by bypassing the heart and lungs, making it a critical therapy for patients in cardiogenic shock [3]. Advances in technology and growing clinical experience have driven a substantial increase in ECMO use, with a reported 400% rise between 2006 and 2011, accompanied by trends toward improved survival and no increase in hospitalization costs [4].

Despite these advances, the rate of complications remains considerable, most notably major bleeding and vascular complications. Consequently, significant efforts have been directed toward identifying risk factors for poor outcomes following ECMO initiation [5]. Importantly, outcomes with VA-ECMO are often influenced by procedural factors - particularly the choice of cannulation strategy. Two principal cannulation approaches are employed in VA-ECMO: peripheral and central. Peripheral cannulation, typically via the femoral vessels, is favored because it can be rapidly performed at the bedside and is less invasive, making it the most widely adopted method worldwide [6]. However, it carries notable risks, most prominently vascular complications, such as limb ischemia [7,8]. Central cannulation, by contrast, involves direct cannulation of the aorta and right atrium, providing more physiologic antegrade flow and potentially superior hemodynamics, but at the cost of greater surgical complexity and higher bleeding risk [8,9]. Thus, the optimal strategy remains uncertain, and practice patterns vary considerably across institutions. Several studies have compared outcomes between peripheral and central VA-ECMO cannulation [9], but findings have been inconsistent. Some highlight bleeding as the predominant complication, whereas others emphasize vascular events, with outcome definitions differing across studies.

To address this gap, we conducted a systematic review and meta-analysis comparing peripheral versus central VA-ECMO cannulation, focusing on major complications, including bleeding, limb ischemia, infection, renal replacement therapy (RRT), and cerebrovascular accidents (CVAs).

## Review

Methodology

Search Strategy and Selection Criteria

This systematic review and meta-analysis evaluate outcomes of peripheral versus central cannulation in VA-ECMO for patients with refractory cardiogenic shock. A comprehensive search of MEDLINE (via PubMed), Scopus, and Cochrane CENTRAL was conducted from database inception through February 2025. Eligible studies enrolled adult patients (≥18 years) treated with VA-ECMO for refractory cardiogenic shock, directly compared peripheral and central cannulation strategies, and reported at least one relevant outcome, including major bleeding, limb ischemia, infection, RRT, or CVA. Both randomized controlled trials and prospective or retrospective cohort studies were included, whereas systematic reviews were screened for additional references but excluded from the quantitative analysis. The Extracorporeal Life Support Organization (ELSO) registry study was excluded to prevent overlap. This review was conducted and reported in accordance with the PRISMA 2020 statement [10].

**Figure 1:**
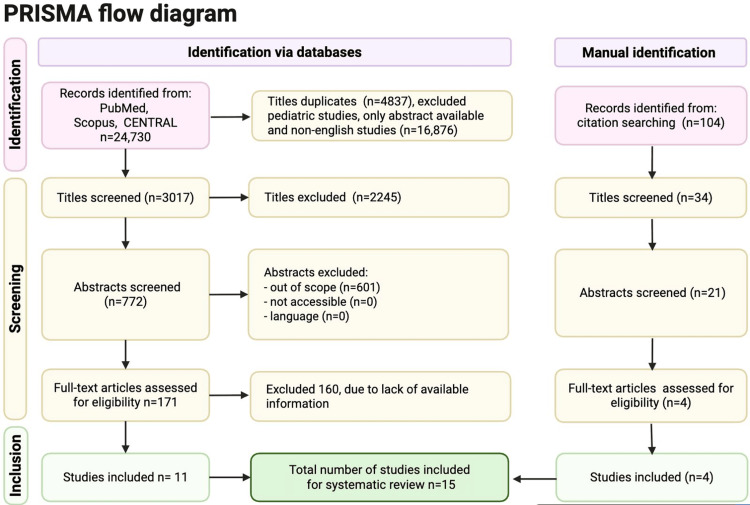
PRISMA flow diagram for study selection: peripheral vs central cannulation in VA-ECMO Database searches of PubMed, Scopus, and CENTRAL yielded 24,730 records. We removed 4,837 duplicates and excluded 16,876 records because they involved pediatric populations, were abstract-only, or were not in English. This left 3,017 titles for screening. We excluded 2,245 records at the title stage and screened 772 abstracts. Of these, 601 abstracts were out of scope, and none were excluded because of inaccessibility or language, leaving 171 full-text articles for eligibility assessment. We excluded 160 full-text articles because of insufficient or unavailable information and included 11 studies from database searches. Citation searching identified 104 additional records. After screening 34 titles and 21 abstracts, four full-text articles met the eligibility criteria. In total, 15 studies were included in the systematic review. Abbreviations: PRISMA, Preferred Reporting Items for Systematic Reviews and Meta-Analyses; ECMO, extracorporeal membrane oxygenation; VA-ECMO, venoarterial ECMO; CENTRAL, Cochrane Central Register of Controlled Trials

Data Extraction and Analysis

Two reviewers independently extracted data from all eligible studies using a standardized form, collecting information on patient characteristics (mean age, sex distribution, baseline comorbidities, and indication for VA-ECMO), intervention details (cannulation strategy, device type, and adjunctive procedures), and clinical outcomes (major bleeding, limb ischemia, infection, RRT, and CVA). Discrepancies were resolved by a third reviewer, and study authors were contacted when essential data were missing.

For each dichotomous outcome, we constructed 2×2 tables and calculated study-specific risk ratios (RRs) with 95% confidence intervals (CIs), comparing peripheral versus central cannulation. Pooled estimates were generated using a DerSimonian-Laird random-effects model, with inverse-variance weighting to account for clinical and methodological heterogeneity. Between-study heterogeneity was assessed with Cochran’s Q test and quantified using the I² statistic, with values of approximately 25%, 50%, and 75% interpreted as low, moderate, and high heterogeneity, respectively. Robustness of the pooled estimates was evaluated through leave-one-out sensitivity analyses, in which each study was sequentially omitted to assess its influence on the overall effect size. All statistical analyses were conducted in R statistical software (v4.3.2, R Foundation for Statistical Computing, Vienna, Austria), using the meta, metafor, and dmetar packages.

Quality Assessment

Using the Newcastle-Ottawa Scale (NOS) for the cohort studies approach to evaluate methodological quality and risk of bias of included studies [11], two reviewers independently assessed the study across multiple variables: (1) Selection; (2) Comparability; (3) Outcome. The scoring system classified studies as high quality, 4-6 as moderate quality, and ≤3 as low quality. Disagreements were resolved through discussion or consultation with a third reviewer. A leave-one-out sensitivity analysis was conducted to assess the robustness of the pooled results (Table 1).

**Table 1:** Summarizes the Newcastle-Ottawa Scale (NOS) quality assessment of the included studies This table summarizes the Newcastle-Ottawa Scale (NOS) quality assessment of the included studies. Each study is evaluated across eight domains (Q1-Q8), with comparability (Q5) weighted up to two points, giving a maximum possible score of 9. The majority of studies scored between 7 and 9 points, indicating overall high methodological quality and low risk of bias. Notably, most studies consistently fulfilled key criteria for representativeness, exposure ascertainment, and outcome assessment. Only a few studies missed points in comparability (Q5) or follow-up adequacy (Q8). Importantly, all studies were categorized as “low risk,” suggesting that the body of evidence included in this analysis is methodologically robust. Q1: Representativeness of exposed cohort; Q2: Selection of non-exposed cohort; Q3: Ascertainment of exposure; Q4: Demonstration that the outcome of interest was not present at the start of the study; Q5: Comparability of cohorts on the basis of the design or analysis (2 points); Q6: Assessment of outcome; Q7: Was follow-up long enough for outcomes to occur; Q8: Adequacy of follow-up of study groups. An overall NOS score of 7 and above is considered low risk, a score of 5 to 6 is considered to have some concerns, and a score under 5 is considered high risk. Sources: [12-26]

Study (Year)	Q1	Q2	Q3	Q4	Q5 (0-2)	Q6	Q7	Q8	Total (0-9)	Risk Category
Ko et al. (2002) [12]	✓	✓	✓	✓	-	✓	✓	✓	7	Low risk
Kanji et al. (2010) [13]	✓	✓	✓	✓	✓	✓	✓	✓	8	Low risk
Mikus et al. (2013) [14]	✓	✓	✓	✓	-	✓	✓	✓	7	Low risk
Loforte et al. (2014) [15]	✓	✓	✓	✓	-	✓	✓	✓	7	Low risk
Saeed et al. (2014) [16]	✓	✓	✓	✓	-	✓	✓	✓	7	Low risk
Khorsandi et al. (2016) [17]	✓	✓	✓	✓	-	✓	✓	✓	7	Low risk
Ranney et al. (2017) [18]	✓	✓	✓	✓	-	✓	✓	✓	7	Low risk
Biancari et al. (2017) [19]	✓	✓	✓	✓	-	✓	✓	✓	7	Low risk
Pichoy et al. (2018) [20]	✓	✓	✓	✓	✓✓	✓	✓	✓	9	Low risk
Mariscalco et al. (2020) [21]	✓	✓	✓	✓	✓✓	✓	✓	✓	9	Low risk
Djordjevic et al. (2020) [22]	✓	✓	✓	✓	-	✓	✓	✓	7	Low risk
Radakovic et al. (2021) [23]	✓	✓	✓	✓	✓	✓	✓	✓	8	Low risk
Saiydoun et al. (2022) [24]	✓	✓	✓	✓	✓	✓	✓	✓	8	Low risk
Alhijab et al. (2023) [25]	✓	✓	✓	✓	✓	✓	✓	✓	8	Low risk
Lee et al. (2024) [26]	✓	✓	✓	✓	✓	✓	✓	✓	8	Low risk

Publication Bias

**Figure 2:**
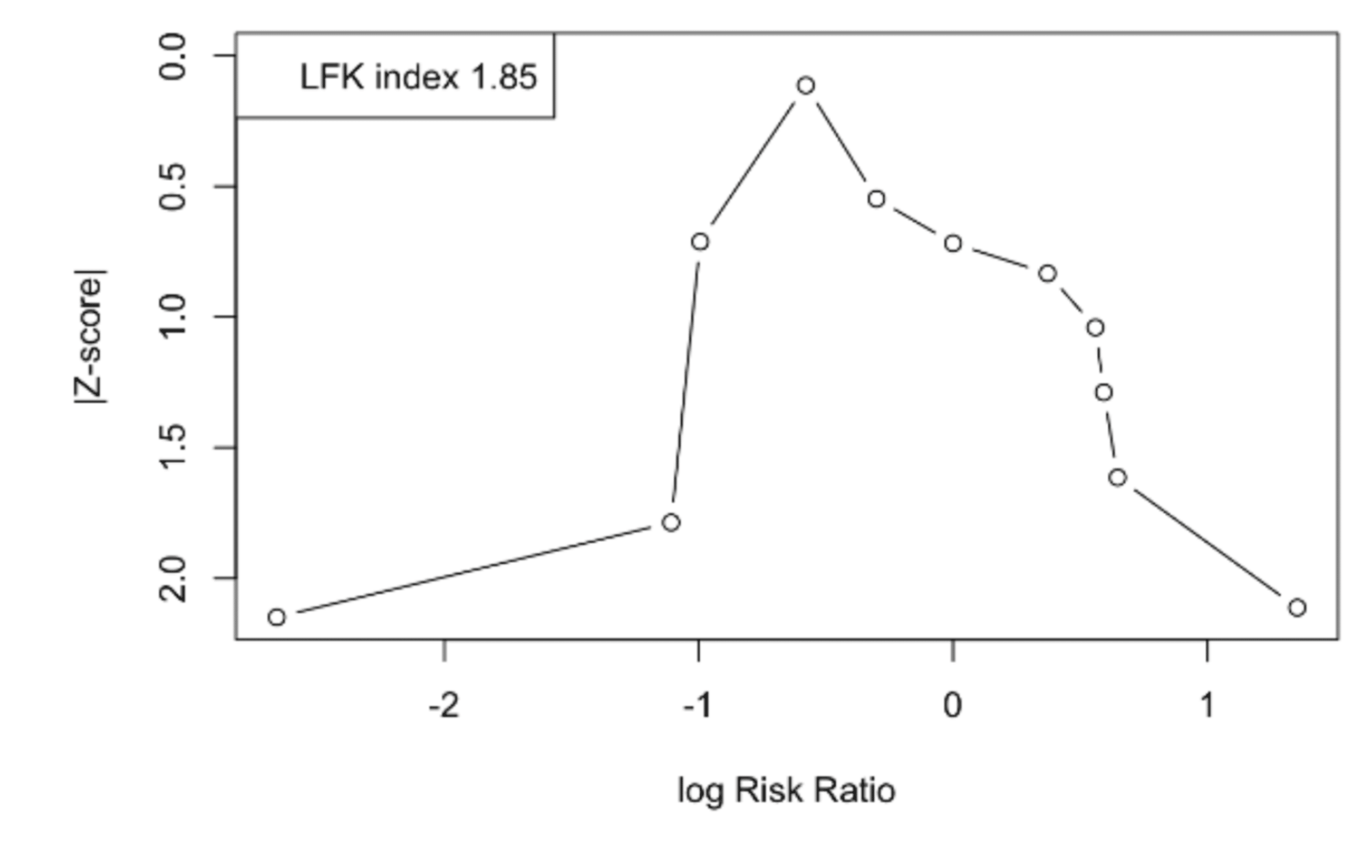
Doi plot The Doi plot illustrates the distribution of study effect sizes (log risk ratios) against their standardized Z-scores, with each circle representing an individual study. The accompanying Luis Furuya-Kanamori (LFK) index is 1.85, which falls between the conventional thresholds of ±1 and ±2, indicating minor asymmetry. This suggests a possibility of small-study effects or publication bias, though the degree of asymmetry is not considered severe. Overall, the findings point to a modest risk of bias in the pooled evidence, warranting cautious interpretation of the meta-analysis results.

Publication bias was evaluated using Doi plots with the Luis Furuya-Kanamori (LFK) index and funnel plots with Egger’s regression test for small-study effects [27,28]. The Doi plot displays the relationship between study effect sizes (log RRs) and their standardized Z-scores, while the LFK index provides a quantitative measure of asymmetry: values within -1 to +1 denote no evidence of asymmetry, values between ±1 and ±2 suggest minor asymmetry, and values ≥±2 indicate major asymmetry, which may reflect selective reporting or small-study effects [28]. Given the modest number of included studies, all assessments of small-study effects were interpreted with caution. The Doi plot illustrates the distribution of study effect sizes (log RRs) against their standardized Z-scores, with each circle representing an individual study. The accompanying LFK index is 1.85, which falls between the conventional thresholds of ±1 and ±2, indicating minor asymmetry [27]. This suggests a possibility of small-study effects or publication bias, though the degree of asymmetry is not considered severe. Overall, the findings point to a modest risk of bias in the pooled evidence, warranting cautious interpretation of the meta-analysis results (Figure 2).

**Figure 3:**
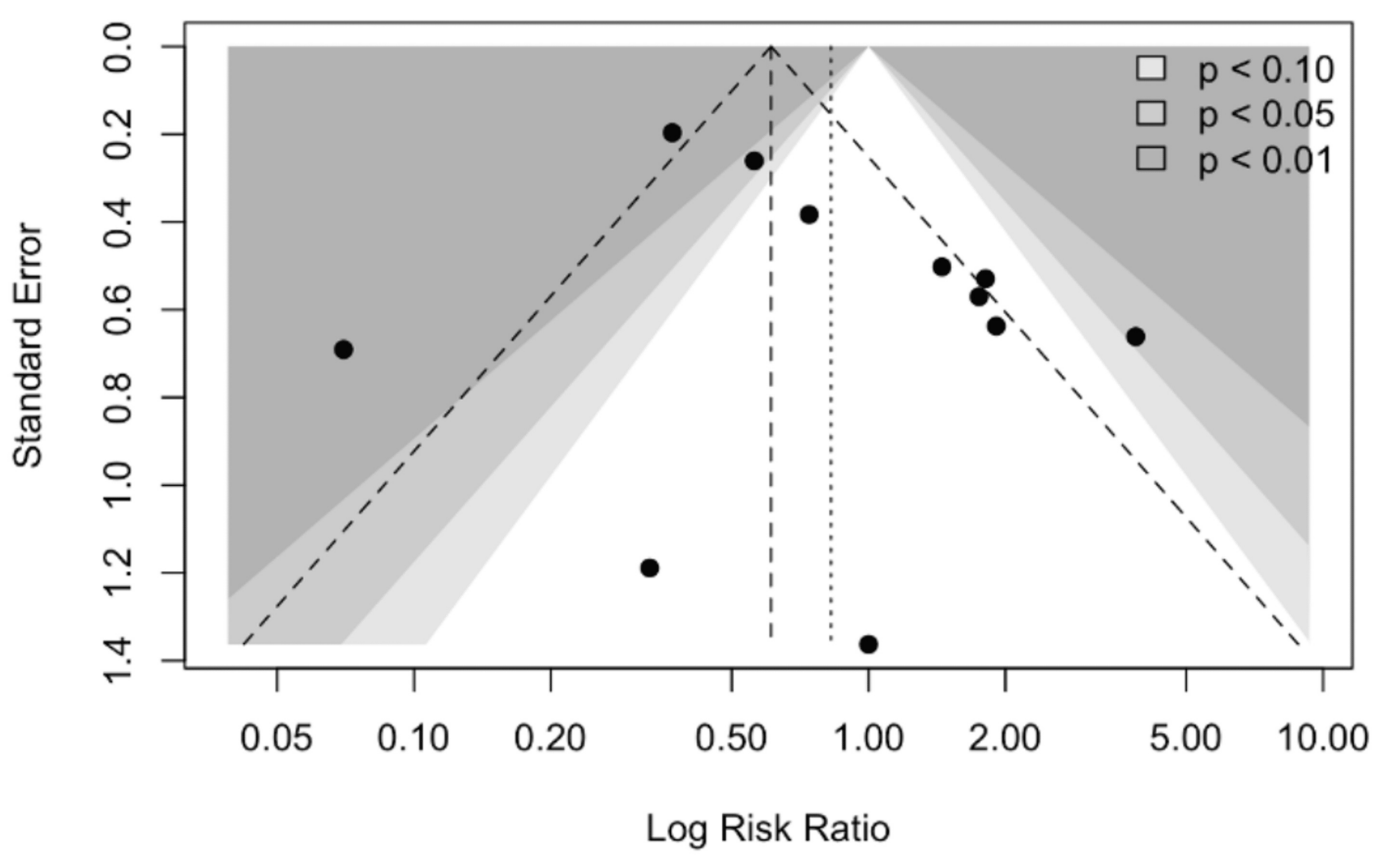
Funnel plot This funnel plot displays the relationship between study effect sizes (log risk ratios on the x-axis) and their precision (standard error on the y-axis). Each black dot represents an individual study, while the shaded triangular regions correspond to levels of statistical significance (p < 0.10, p < 0.05, and p < 0.01). In the absence of publication bias, studies are expected to be symmetrically distributed around the pooled estimate (vertical dashed line) within the funnel. In this plot, the distribution appears somewhat asymmetric, with a clustering of smaller studies showing larger effect sizes on one side. This pattern raises the possibility of small-study effects or publication bias, although some asymmetry may also be explained by heterogeneity across studies.

In the funnel plot, the distribution appears somewhat asymmetric, with a clustering of smaller studies showing larger effect sizes on one side. This pattern raises the possibility of small-study effects or publication bias, although some asymmetry may also be explained by heterogeneity across studies (Figure 3).

**Figure 4:**
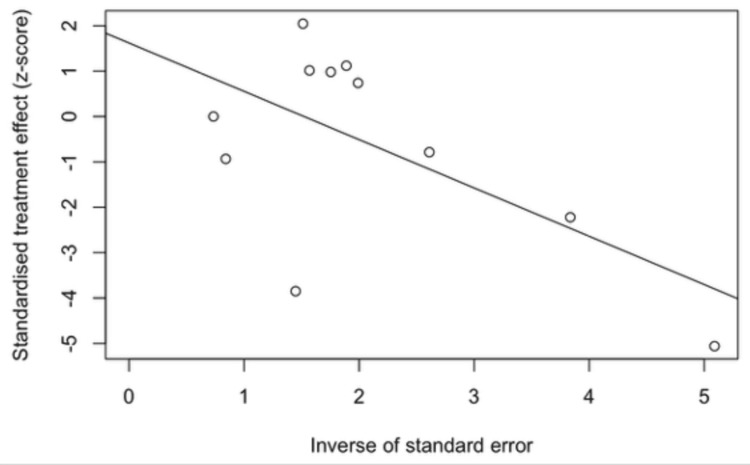
Egger’s regression plot for small-study effects This plot represents an Egger’s regression test for small-study effects, a method commonly used to assess publication bias in meta-analysis. The x-axis shows the inverse of the standard error (a measure of study precision), while the y-axis shows the standardized treatment effect (Z-score). Each open circle corresponds to an individual study, and the fitted regression line evaluates the relationship between effect size and study precision. In the absence of publication bias, the regression line should be nearly horizontal, indicating no systematic association. Here, the downward-sloping line suggests a potential asymmetry: smaller, less precise studies may be reporting disproportionately larger or more variable effects. This pattern indicates possible publication bias or small-study effects, though heterogeneity may also contribute.

Egger’s regression test pattern indicates possible publication bias or small-study effects, though heterogeneity may also contribute (Figure 4) [28]. 

Results

Study Selection

Database searches of PubMed, Scopus, and CENTRAL yielded 24,730 records. After removing 21,713 records - comprising 4,837 duplicates and exclusions for pediatric populations, abstract-only reports, and non-English articles - 3,017 titles were screened. Of these, 2,245 were excluded at the title level, leaving 772 abstracts for review; 601 were out of scope, and none were excluded for inaccessibility or language. Fourteen full texts were assessed, with three excluded for insufficient information, resulting in 11 eligible studies from database searches. Citation searching identified 104 additional records; 34 titles and 21 abstracts were screened, and four full texts met eligibility. In total, 15 studies were included in the systematic review (Figure 1).

Study Characteristics and Quality Assessment

Fifteen studies (study periods 2000-2024) met inclusion, encompassing nearly 3,000 adults supported with VA-ECMO for cardiogenic shock. Cohorts were predominantly post-cardiotomy CS, with several mixed-etiology CS series and one ECPR study. Sample sizes ranged from 14 to 814 (median, 120). Reported ages clustered in the late 50s to mid 60s, and populations were predominantly male (typically ~65%-70%). Where available, surgical risk was high: EuroSCORE II medians ~7.5-11 and means up to ~19%, with wide ranges for logistic EuroSCORE. Detailed demographics and baseline characteristics are presented in Table 2.

**Table 2:** General review of the manuscripts Characteristics and key outcomes of studies included in the meta-analysis of peripheral versus central cannulation in VA-ECMO. The table summarizes, for each study, the design, setting, study period, sample size, baseline demographics, and primary indications for VA-ECMO or ECLS. It also reports main clinical outcomes, including survival, bleeding and vascular complications, limb ischemia, need for renal replacement therapy, re-exploration for bleeding, longer-term functional status, and resource use, as well as preoperative risk scores, where available (logistic EuroSCORE, EuroSCORE II). Abbreviations: VA-ECMO, venoarterial extracorporeal membrane oxygenation; ECMO, extracorporeal membrane oxygenation; ECPR, extracorporeal cardiopulmonary resuscitation; PCCS, post-cardiotomy cardiogenic shock; CABG, coronary artery bypass grafting; CPB, cardiopulmonary bypass; ICU, intensive care unit; NYHA, New York Heart Association; RRT, renal replacement therapy; FFP, fresh frozen plasma; V-A ECLS, venoarterial extracorporeal life support; LV, left ventricle; VAD, ventricular assist device; AMI, acute myocardial infarction; HF, heart failure; CS, cardiogenic shock; CVVH, continuous veno-venous hemofiltration; CK-MB, creatine kinase MB isoenzyme; PRBC, packed red blood cells; FiO₂, fraction of inspired oxygen; PO₂, partial pressure of oxygen; PCO₂, partial pressure of carbon dioxide Sources: [12-24]

Study (Author, Year) and Design/Setting	Study Period	Sample Size (N)	Baseline Characteristics (Age, Sex)	Indication/Population	Key Outcomes and Complications	Risk/Severity Score (EuroSCORE, Logistic EuroSCORE, etc.)
Khorsandi et al (2016); multicenter retrospective study (Scotland)	April 1995 - April 2015	27	Median 59 years; 85% male	Refractory post‐cardiotomy cardiogenic shock (PCCS)	Survival to discharge: 40.7%; complications: major hemorrhage, renal failure (RRT), fatal stroke, septic shock, femoral pseudoaneurysm; survivors NYHA I–II at 12 months	Logistic EuroSCORE ranged from 2.08 to 73.26 (no single mean value reported)
Kanji et al (2010); single‐center retrospective analysis	January 2002 - December 2006	50	Demographics are similar between groups (not explicitly detailed)	Post‐cardiotomy ECMO support for cardiac surgery patients	Limb ischemia incidence was similar; central cannulation had higher bleeding from the cannulation site (64% vs. 18%), greater blood product use and reoperation rates; 30‐day mortality was similar (46% peripheral, 50% central)	Not reported
Lee et al (2024); single‐center retrospective study (ECPR)	January 2017 - May 2023	99	Mean age ~59 years (similar between groups)	Extracorporeal cardiopulmonary resuscitation (ECPR) in cardiac arrest	Percutaneous cannulation significantly shortened ECMO insertion time; low‐flow time was significantly associated with mortality; survival to discharge was ~30-36%; no significant difference in survival between the surgical and percutaneous groups	Not reported
Djordjevic et al. (2020); retrospective analysis	April 2006 - October 2016	156	cECMO: ~65 yrs; pECMO: ~61 yrs (trend toward younger in peripheral)	Postcardiotomy cardiogenic shock	30‐day mortality ~70% in both groups; central cannulation had higher rates of on‐site complications (bleeding, re‐exploration, FFP transfusions); after matching, no significant differences in mortality	Median EuroSCORE II: ~9-11 (Central: 11 (IQR 7.5-14) vs. Peripheral: 9 (IQR 6-12))
Biancari et al. (2017); multicenter study	2005 - 2016	148	Patients were older (exact mean not provided)	Post-CABG ECMO for cardiac/respiratory failure after CABG	In‐hospital mortality 64.2%; predictors included lower creatinine clearance, presence of pulmonary disease, higher lactate; 1-, 2-, and 3-year survival: 31%, 27.9%, 26.1% respectively	Mean EuroSCORE II: 19.2%
Mikus et al. (2013); retrospective observational study	January 2007 - August 2011	14	Mean age: 53.1 ± 14.3 yrs; 64.3% male	Postcardiotomy cardiogenic shock (failure to wean from CPB/within 48h in ICU)	50% weaned from ECMO; 42.9% died on support; 42.8% discharged home; Median support duration: 5 days	Not reported
Pichoy et al. (2018), retrospective propensity score-matched study	January 2015 - December 2017	814 total (485 surgical, 329 percutaneous; 266 matched pairs)	Surgical group: ~53.6 ± 14.3 yrs; Percutaneous: ~55.8 ± 14.5 yrs; ~70% male (≈30% female)	VA‑ECMO for refractory/postoperative cardiogenic shock	Percutaneous cannulation is associated with fewer local infections (16.5% vs. 27.8%), similar limb ischemia rates, and improved 30‑day survival (63.8% vs. 56.3%), though with higher vascular complications after decannulation	Not reported
Mariscalco et al. (2020); multicenter registry, systematic review & meta‑analysis)	January 2010 - March 2018	781	Mean age: 63.1 ± 12.9 yrs; 32% female	Postcardiotomy shock (38% failure to wean from CPB; 48% heart failure after CPB)	Meta‑analysis pooled in‑hospital/30‑day mortality: 66.6%; Central cannulation associated with higher mortality, more re‑operations for bleeding, and greater transfusion needs compared to peripheral cannulation	EuroSCORE II ≈9 (range ~3.4-26.8)
Radakovic et al. (2021); observational retrospective single-center analysis	January 2010 - January 2019	158	Mean age ≈66 years; ~72% male, ~28% female	VA-ECMO for postcardiotomy cardiogenic shock	Cannulation‐related complications (arterial site bleeding, surgical revision, Harlequin’s syndrome); LV unloading; ECMO weaning rate; 30‑day and in‑hospital mortality; additional postoperative events	Log EuroSCORE I: Central group: 41.9 ± 20; Peripheral group: 44.3 ± 27.7
Ranney et al. (2017); retrospective single-center study	June 2009 - April 2015	131	Mean age 56.4 ± 13.5; Male 67.9%, Female 32.1%	VA-ECMO for various indications - predominantly cardiogenic shock (93.1%), with some respiratory failure and others	Overall vascular complications 22.1% (including cannula re‐location and site bleeding variations); median ECMO duration 4 days; median hospital length of stay 22 days; in‐hospital mortality 58% (similar survival across cannulation types)	Not reported
Saeed et al. (2014); retrospective study comparing pECMO vs. cECMO	October 2009 - June 2011	37 (25 pECMO; 12 cECMO)	pECMO: 59 ± 16 years; cECMO: 70 ± 5 years	pECMO: mixed indications (≈52% postcardiotomy); cECMO: all postcardiotomy	Immediate postimplantation hemodynamics and end‐organ parameters were similar; at 3 hours, pECMO showed higher PO₂ and lower PCO₂ (likely due to higher FiO₂); re‐exploration for bleeding in 44% (pECMO) vs. 100% (cECMO); 30‐day mortality 60% vs. 67%; overall, less bleeding/resternotomy complications with pECMO	Not reported
Saiydoun et al. (2022); prospective observational registry	March 2018 - March 2021	120	Median age 57 (47-66) years; 72% male overall; surgical group: 53 (41-64) vs. percutaneous group: 60 (51-67) years (p = 0.007)	Peripheral femoro‐femoral V-A ECLS for refractory cardiogenic shock or cardiac arrest (non‐postcardiotomy)	Angio-guided percutaneous approach associated with a higher weaning rate (46% vs. 29%, p = 0.05) and significantly fewer major vascular complications (e.g., major bleeding 7% vs. 31%, p = 0.0007); 30‐day survival rates were similar (26% vs. 17%, p = 0.22)	Not reported
Alhijab et al. (2023); retrospective cohort study	June 2009 - December 2020	101	Median age: ~60 years (Central: 62.5; Peripheral: 58); overall ~57% female	Postcardiotomy cardiogenic shock	Peripheral ECMO was associated with higher limb ischemia; bleeding/transfusion requirements were similar between groups; central cannulation yielded better 1‑year survival and resource utilization; predictors of mortality included age, cannulation type, and infective endocarditis	Median EuroSCORE II ~7.5-9.3 (reported separately for two groups)
Loforte et al. (2014); double-center retrospective study	January 2006 - December 2012	228	Mean age: 58.3 ± 10.5 years; 155 men (67.9%) (range: 19–84 years)	Refractory cardiogenic shock: postcardiotomy (n = 118), primary donor graft failure (n = 37), post-AMI CS (n = 27), acute myocarditis (n = 6), and chronic HF CS (n = 40)	Mean ECMO support time 10.9 ± 9.7 days; ECMO mortality 36.8%; overall success rate (weaning, bridge to VAD (2.6%), bridge to heart transplant (13.5%)) 63.1%; 53.5% of patients discharged; logistic regression identified blood lactate level and CK-MB relative index at 72 h plus PRBC transfusions as significant predictors of ECMO mortality; central ECMO had higher rates of CVVH and bleeding requiring surgery; persistent LVEF ≤40% predicted late death.	Not reported
Ko et al. (2002); retrospective review	August 1994 - May 2000	76	Mean age 56.8 ± 15.9 years; 48 men (63%) and 28 women (37%)	Postcardiotomy cardiogenic shock	30 died on ECMO; 22 weaned but died in hospital; 20 weaned and survived to discharge (≈28% survival)	Not reported

Association of ECMO and Outcomes

**Figure 5:**
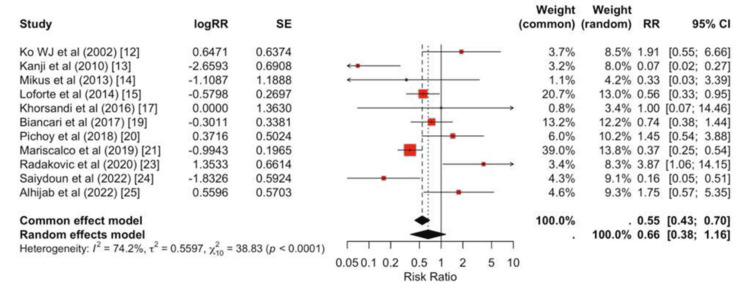
Bleeding forest plot Random-effects forest plot of bleeding comparing groups across individual studies, with study-specific effect sizes and 95% CIs shown as squares and lines, and the pooled estimate as a diamond. The summary effect favors the exposure associated with lower bleeding risk (pooled RR < 1), with low-to-moderate between-study heterogeneity.

Peripheral cannulation was associated with a significantly lower risk of major bleeding compared with central cannulation (pooled RR = 0.55, 95% CI, 0.43-0.70; Figure 5).

**Figure 6:**
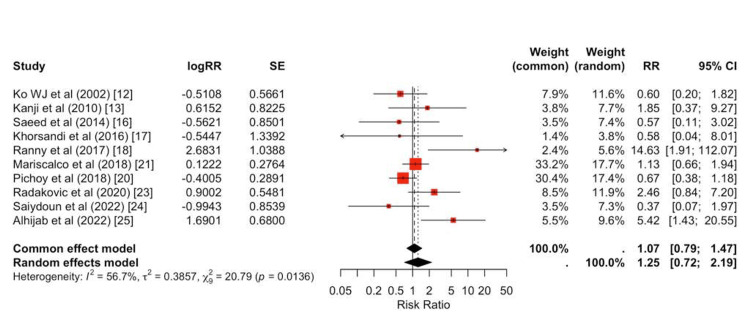
Forest plot for limb ischemia Random-effects forest plot for limb ischemia comparing cannulation strategies. Most studies lie to the right of unity, indicating a higher risk of limb ischemia with peripheral cannulation. The pooled effect is elevated (RR > 1), with moderate heterogeneity (I² ~55%-60%).

In contrast, peripheral cannulation was associated with a higher risk of limb ischemia (pooled RR = 1.43, 95% CI, 1.17-1.75; Figure 6).

**Figure 7:**
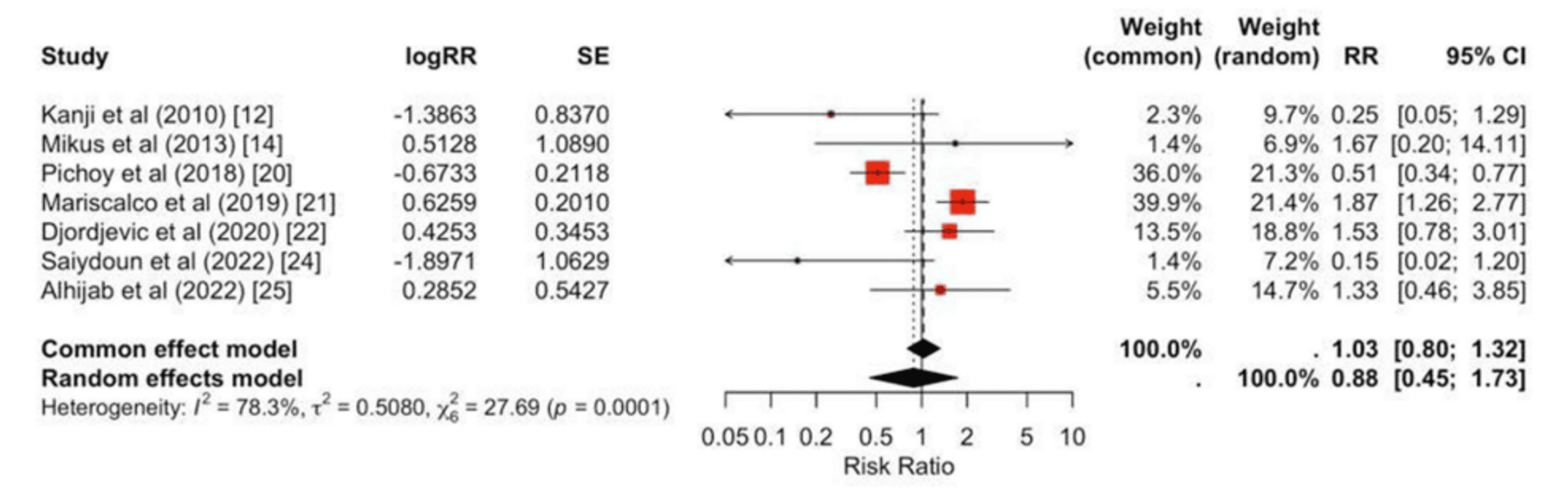
Forest plot for infection Individual study RRs span both sides of unity, and the pooled effect is null (RR ≈ 1.03, 95% CI 0.80-1.32), indicating no statistically significant difference in infection between groups, and between-study heterogeneity is substantial (I² ≈ 78%).

**Figure 8:**
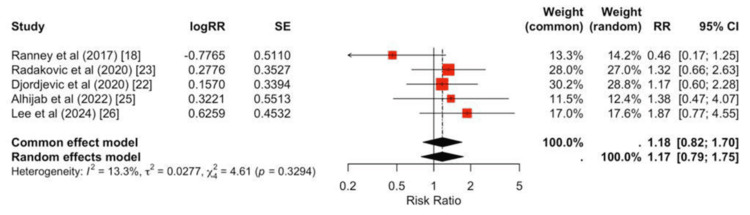
Forest plot for renal replacement therapy Study-specific RRs span both sides of unity, and the pooled effect is not significant (RR ≈ 1.18, 95% CI 0.82-1.70), with low heterogeneity (I² ≈ 13%).

**Figure 9:**
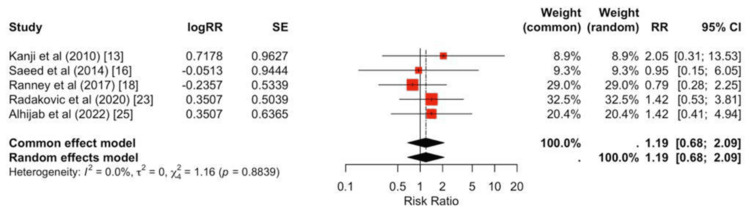
Forest plot for cerebrovascular accidents Study estimates cluster around unity, and the pooled effect is not significant (RR ≈ 1.19, 95% CI 0.78-1.83). Between-study heterogeneity is negligible (I² = 0%).

No significant differences were observed between peripheral and central strategies for infection (pooled RR = 0.88, 95% CI 0.39-2.01; Figure 7), RRT (pooled RR = 1.17, 95% CI 0.66-2.08; Figure 8), or CVA (pooled RR = 1.19, 95% CI 0.78-1.83; Figure 9). 

Discussion

This meta-analysis provides robust evidence on the procedural trade-offs between peripheral and central cannulation for VA-ECMO in refractory cardiogenic shock. In nearly 9,000 patients and 15 studies, peripheral cannulation consistently reduced the risk of major bleeding but increased the incidence of limb ischemia, while systemic outcomes such as infection, RRT, and CVA showed no consistent differences. 

This meta-analysis confirms that the most consequential procedural hazards of VA-ECMO lie in bleeding and vascular complications, with distinct risk profiles for central and peripheral cannulation. Central access consistently carried a greater risk of bleeding, driven by the inherent surgical exposure of sternotomy or thoracotomy, the use of larger cannulae, and the friable tissue milieu of postcardiotomy patients. Early series reported mediastinal bleeding as a dominant complication, with re-exploration rates exceeding 40% in central ECMO cohorts [12,13]. Subsequent single-center experiences reproduced this gradient, showing that mediastinal oozing, tamponade, and re-exploration substantially increased transfusion burden and infectious risk [15,18]. Contemporary reports, incorporating percutaneous central techniques, heparin-bonded circuits, and restrictive transfusion thresholds, demonstrated attenuation of bleeding but did not eliminate the excess, underscoring that the invasive surgical substrate remains the principal driver [20,21]. Large registry analyses corroborate these findings, consistently associating central cannulation with increased hemorrhagic morbidity and transfusion requirements compared with peripheral access [29,30].

Peripheral cannulation, in contrast, imposes a vascular penalty, most notably limb ischemia. The femoral route redirects antegrade arterial flow, creating risk of distal hypoperfusion, embolic phenomena, dissection, and, in severe cases, compartment syndrome. Across studies, ischemic complication rates demonstrated wide variation, reflecting differences in sheath-to-artery ratio, cannula size, operator expertise, and institutional protocols. The absence of distal perfusion catheters (DPCs), reliance on emergent insertion without imaging guidance, and lack of structured vascular surveillance were consistent predictors of ischemia [18,31]. Conversely, standardized DPC placement, ultrasound guidance, and formal monitoring protocols markedly reduced ischemic complications, although adoption remained inconsistent across centers [16]. Despite these refinements, contemporary practice continues to demonstrate that peripheral VA-ECMO carries a measurable ischemic penalty, compared with central access.

Collectively, these findings illustrate a clear trade-off: central cannulation exposes patients to higher hemorrhagic morbidity, due to the invasiveness of surgical exposure and mediastinal bleeding, whereas peripheral cannulation carries an increased risk of vascular compromise, related to cannula-artery interactions and heterogeneity in adjunctive protective measures. Systemic outcomes, such as infection, cerebrovascular events, and RRT, have shown inconsistent patterns across the literature, but access-related bleeding and ischemia remain the dominant procedural complications. These observations underscore the need for strategy-specific vigilance: rigorous hemostatic and transfusion protocols in central ECMO, and structured vascular protection bundles in peripheral ECMO. As modern ECMO practice shifts toward rapid percutaneous deployment, balancing these access-related hazards remains central to optimizing patient outcomes.

Limitation

Limitations of the available literature include retrospective design, heterogeneous case-mix, evolving anticoagulation protocols, and non-standardized outcome definitions. Central ECMO is often reserved for postcardiotomy or ECPR patients, with intrinsically higher risk, complicating direct comparisons. Long-term survival data are sparse, and key complications, such as ischemia, bleeding, or stroke, are variably adjudicated. 

Several limitations should be considered when interpreting this meta-analysis. Most included studies were retrospective and observational, introducing risks of selection bias, reporting bias, and residual confounding. Central cannulation was frequently applied in postcardiotomy or extracorporeal cardiopulmonary resuscitation (ECPR) settings, where patients were intrinsically sicker, with higher lactate, greater vasopressor requirements, and worse pre-ECMO physiology. This indication bias complicates direct comparisons with peripheral cohorts and likely amplifies differences in bleeding, ischemia, or survival outcomes.

The absence of standardized outcome definitions and device-level data limits causal interpretation and prevents adjustment for technical factors known to influence complications.

Follow-up duration was limited in most studies, with outcomes generally restricted to in-hospital or 30-day endpoints. Long-term survival, quality of life, and functional recovery remain poorly characterized. Registry data suggest that only a minority of survivors achieve durable recovery, yet these outcomes were rarely reported in single-center or smaller series. Consequently, this analysis cannot assess the durability of survival or late complications such as chronic renal dysfunction, neurocognitive decline, or vascular sequelae. Survivor bias adds further complexity: patients who died early were unlikely to develop complications such as infection or limb ischemia, leading to underestimation of event rates in sicker central cohorts.

Publication bias cannot be excluded. Funnel plots and Egger’s regression did not reveal significant small-study effects for bleeding, yet the number of available studies was modest, and negative or neutral findings may remain unpublished. Moreover, outcomes reported by high-volume ECMO centers may not reflect those in lower-volume or resource-limited settings, limiting generalizability. Outcomes are also shaped by institutional expertise, operator skill, and standardized protocols (e.g., structured vascular surveillance, shock-team activation, and decannulation strategies). Most studies did not adjust for these volume-outcome relationships, further constraining external validity.

Despite these limitations, the consistent signal of reduced bleeding with peripheral access supports its clinical relevance. Future prospective, multicenter, and device-aware studies are needed to refine patient selection, optimize anticoagulation and transfusion strategies, and assess the long-term impact of limb ischemia. In parallel, pragmatic registries with uniform outcome definitions and real-time data capture may represent the most feasible path forward, given the challenges inherent in conducting adequately powered, randomized trials in this critically ill population.

Clinical implication

The cumulative evidence is pragmatic and consistent: peripheral cannulation remains the preferred strategy in most cases because of lower bleeding risk, rapid initiation, and ease of use, but it requires vigilant ischemia prevention through DPCs and structured surveillance. Central cannulation remains critical in postcardiotomy shock or when antegrade flow is prioritized, at the expense of higher bleeding and re-exploration risk.

The findings of this meta-analysis have several practical implications for the management of patients requiring VA-ECMO. First, peripheral cannulation should generally be considered the default strategy due to its lower risk of major bleeding and ease of initiation, especially in time-sensitive settings such as cardiogenic shock or ECPR. This approach may reduce transfusion requirements, minimize re-exploration, and improve hemodynamic stability in critically ill patients.

Additionally, the increased risk of limb ischemia associated with peripheral access highlights the importance of structured preventive protocols, including the routine use of DPCs, ultrasound-guided cannulation, and standardized vascular monitoring. Institutions with formal limb-protection strategies are more likely to achieve favorable outcomes without sacrificing the bleeding advantage of peripheral access.

Third, central cannulation should remain reserved for selected patients, such as those with severe peripheral arterial disease, postcardiotomy shock, or situations where direct cardiac decompression is required. In these scenarios, the bleeding risk is offset by the hemodynamic benefits of antegrade flow and direct ventricular unloading.

Finally, optimal outcomes with either strategy depend not only on the choice of cannulation site but also on institutional expertise and perioperative protocols. Anticoagulation management, transfusion thresholds, and early detection of complications are critical determinants of success and should be integrated into standardized care pathways.

## Conclusions

This meta-analysis demonstrates that peripheral VA-ECMO cannulation is associated with a significantly lower risk of major bleeding compared with central cannulation, while carrying a higher risk of limb ischemia. Other complications, including infection, the need for RRT, and CVAs, did not differ meaningfully between strategies. Therefore, the choice between peripheral and central access should be individualized based on patient-specific risk profiles, particularly by balancing bleeding risk against ischemic risk.
